# The IAEA’s activities on radiation protection in interventional cardiology

**DOI:** 10.2349/biij.3.2.e31

**Published:** 2007-04-01

**Authors:** MM Rehani

**Affiliations:** International Atomic Energy Agency, Vienna, Austria

**Keywords:** Training in radiation protection, cardiac catheterization- radiation protection, IAEA training, radiation protection in interventional cardiology

## Abstract

The International Atomic Energy Agency (IAEA) under its mandate of developing and applying standards of radiation safety has initiated a number of activities in recent years on radiation protection in interventional cardiology. These activities are implemented through four mechanisms, namely training, providing information through the website, research projects and assistance to Member States through Technical Cooperation (TC) projects. Major international initiatives have been taken in the area of training where more than half a dozen regional training courses have been conducted for cardiologists from over 50 countries. Additionally four national training events for over 300 medical and paramedical staff members involved in interventional procedures were held. The training material is freely available on CD from the IAEA. The newly established website provides information on radiation protection issues [1]. Two coordinated research projects have just been completed where peak skin doses to patients undergoing high dose interventional procedures were studied and factors to manage patient doses were identified. The technical cooperation projects involving protection in cardiac interventional procedures have 30 countries as participants.

## INTRODUCTION

The number of interventional cardiological procedures continue to increase at a steep rate in most countries. As a result, cardiologists employ a significant amount of radiation in most cases that can be matched only with interventional procedures performed by radiologists. While interventional radiologists normally undergo training in radiological physics and radiation protection (RP&RP) during their residency, the same is not true of cardiologists. In most countries cardiologists have no training in RP&RP. In previous years, typically in the late 1970’s and early 1980’s when the interventional era started, radiologists either performed the cardiac interventional procedures or were associated with the procedures. Over the years cardiologists have acquired independent catheterization laboratories (cath labs) but have not been trained in the same way as radiologists. This creates definite radiological protection problems for patients and for cardiologists themselves [2,3]. A number of radiation induced skin injuries have been reported in patients undergoing cardiac angioplasty and other interventions [2]. When cases of radiation injuries were brought to the courts, the cardiologist invariably had no knowledge about the possibility of such injuries, as their training did not cover radiation effects. Further, RP&RP is hardly taught in medical schools during undergraduate studies. Medical professionals who undertake further education in radiological specialties such as diagnostic radiology, nuclear medicine and radiotherapy are taught RP&RP studies.

The International Basic Safety Standards (known as BSS) for Protection against Ionizing Radiation and for the Safety of Radiation Sources, published by the International Atomic Energy Agency (IAEA) and jointly sponsored by FAO, ILO, PAHO and WHO [4], require that all personnel whose work has implications on protection and safety be appropriately trained and qualified so that they understand their responsibilities and perform their duties with appropriate judgment and according to defined procedure. The BSS also requires that the training criteria be specified.

Realizing these lacunae in effective training, the IAEA has initiated actions to create awareness among interventional cardiologists on risks to patients and staff in fluoroscopic angiographic procedures performed by them.

## TRAINING

Recommendations for training have emerged from various national and international sources [5-10]. Most of these are recommendations, consensus statements or are derived from general requirements or Directives [11]. The IAEA has developed a curriculum with educational objectives specifically for interventional cardiologists (Table 1). A review of recommendations on training from different sources can be found in a recent publication by Rehani [3]. It is aimed primarily at developing countries where cardiology professional societies are not yet sufficiently advanced to undertake separate modules for basic and advanced curricula in the field of RP. For such countries, a simple module is ideal, particularly in view of the lack of experts in RP&RP in diagnostic imaging to teach the subject to cardiologists. The IAEA has also prepared educational material in the form of PowerPoint slides on CD entitled ‘IAEA Training Material on Radiation Protection in Cardiology’. The material is available free of charge and can be obtained by writing to patient.protection@iaea.org; and will in future be available for download from the website [1]. Based on this training material, the IAEA has conducted a large number of training courses (Tables 2 and 3). The feedback from the participants is given in Table 3. It is quite evident that training in radiation protection has been inadequate in most countries and the need for formal training is felt across the board and includes training for national requirements within a legislative framework. The survey also included feedback on factors such as length of the course, suitability of the curriculum and further recommended actions. 92% of the participants in Vienna and 96% in the Singapore meeting stated that the course length of two days was suitable. The curriculum was, by and large, satisfactory, only very few participants mentioned that it was slightly too technical. The almost unanimous opinion was that such programmes should be conducted more often and in many countries, papers on RP should be published in cardiology journals and RP lectures should be included in cardiology conferences.

**Table 1:** IAEA Training Curriculum on Radiation Protection in Cardiology

**S.No.**	**Topic**	**Educational objectivesAt the end of the programme, the participants should know these**
1	Why talk about radiation protection in cardiology?	a. Review of severity and frequency of radiation injuries b. What do these injuries teach us regarding the cardiologist’s role (Lessons learned)? c. Points-of-view about law suits of severe injury d. Recognizing radiation injury and effects
2	Talking about radiation dose	a. How radiation dose can and should be expressed: merits and demerits of each quantity for cardiology practice b. How representative fluoroscopy and cine times are for dose to the patient and staff c. Simplified presentation of radiation quantities
3	What radiation effects are possible (besides skin injuries)?	a. Understanding about stochastic and deterministic effects b. Probability of these effects in interventional practice c. Special concerns in children, young females and pregnant women
4	Angiography equipment	a. What are equipment standards for cath equipment (FDA, IEC), particular needs for paediatric patients b. Comparative features of available angiography equipment c. Dose variations in cine and fluoro modes
5	Patient dose management – equipment and physical factors	a. Physical factors and challenges in dose management b. Understanding the role of operator in patient dose management c. How to manage patient dose using equipment factors
6	Standards and guidance	a. Standards and guidance provided by international organizations b. Who is responsible for what? c. What actions are needed by cardiologists
7	Occupational exposure and protective devices	a. How effective are individual protective items in cath lab b. How to monitor personnel dose c. How to estimate personnel effectiveness
8.1	Image quality in cardiac angiography	a. How can image quality of cardiac angiographic images be assessed b. How useful can the quality criteria be
8.2	Impact of optimization in newer technologies	a. What benefit of digital flat panel technology can be expected on patient dose? b. How to translate this into practice c. Experience with optimization
8.3	Can cardiac procedures be graded in complexity and related with dose?	a. Complexity related factors in cardiac interventions b. Relationship of patient dose with technical and clinical factors c. Development of complexity index and its utility
9.1	Examples of good and bad practice	a. How wedge filter and field size affect skin dose b. When and how to use the wedge filter
9.2	Example of practice of radiation protection	a. How awareness of radiation protection and close cooperation with medical physics/ radiation safety staff helps b. Avoidance of skin injuries
10	Radiation risks in paediatric interventional cardiology	a. Unique considerations in paediatric patients having bearing on patient dose b. How can dose be managed in paediatric patients

**Table 2:** List of regional training courses on radiation protection in cardiology and countries from where cardiologists participated

**Year**	**Course Location**	**Participating Cardiologists from **
May 2004	Vienna	**East Asia: **Bangladesh, China, Indonesia, Malaysia, Mongolia, Myanmar, Pakistan, Philippines, Singapore, Sri Lanka, Thailand **West Asia:** Iran, Kazakhstan, Lebanon, Syria, Tajikistan, Yemen **Europe:** Albania, Bulgaria, Latvia, Lithuania, Moldova, Poland, Romania
Apr 2005	Singapore	Bangladesh, India, South Korea, Myanmar, Singapore, Thailand, Vietnam
Apr 2006	Chile	Argentina, Bolivia, Brazil, Chile, Columbia, Cuba, Ecuador, Guatemala, El Salvador, Nicaragua, Paraguay, Uruguay
May 2006	Ethiopia	Algeria, Democratic Republic of Congo, Ethiopia, Kenya, Mali, Sudan, Tunisia, Tanzania, Zimbabwe.
Aug 2006	Iran	Afghanistan, Iran, Jordan, Qatar and United Arab Emirates (UAE)
Dec 2006	Thailand	Bangladesh, China, Indonesia, Malaysia, Mongolia, Singapore, Thailand, Vietnam
**National Training courses with participation of 80-150 (in each course) cardiologists, radiographers and nurses**		
Dec 2005	Thailand	
Apr 2006	Kuala Lumpur	
Sep 2006	Philippines	
Nov 2006	Sri Lanka	
Jan 2007	Egypt	

**Table 3:** Feedback from participants in the regional training courses

	**Vienna** **2004**	**Singapore** **2005**	**Ethiopia** **2006**	**Iran** **2006**	**Bangkok** **2006**
Is this the 1st time you are attending a structured program on RP? (Ans.: YES)	88%	84%	93%	100%	93%
Any cardiologists’ conference you attended where there was lecture on RP? (Ans.: NO)	85%	100%	100%	100%	100%
Do you measure radiation dose to the patient? (Ans.: NO)	96%	100%	87%	89%	71%
Which are the top two professionals likely to get higher radiation exposure in normal practice?	50%	11%	20%	44%	50%
Do you use a badge to monitor your personal exposure? (Ans.: YES)	77%	74%	33%	48%	57%

To consolidate the gains and maintain the momentum generated by training, the IAEA has initiated a project under a Regional Cooperation Agreement (RCA) for Asia to create a network of cardiologists who will continue radiation protection in their countries through the national and regional cardiological societies. The group of cardiologists trained by the IAEA would meet every year and develop strategies and action plans. The project started in 2007 and the first meeting of the network was held in Sarawak, Malaysia, wherein it was decided to start an e-newsletter soon. This will, in all likelihood, be the very first newsletter by cardiologists in the area of radiation protection. The network of Asian cardiologists shall organize sessions on radiation protection in various cardiology conferences in the region.

How much training is adequate? This issue has been addressed in a recent publication by the author of this paper [3]. To quote from this paper “Since the intensity of radiation employed by interventional cardiologists per patient, and collectively based on workload, is no less than that employed by interventional radiologists, the training standards of RP&RP in interventional cardiology should also match those in interventional radiology. However, this may be practically impossible to achieve, much as it is desired. Attempts should be made to begin at least with what is practicable and achievable. This stems from the fact that interventional cardiology may consist of just 5-20% of the work of many cardiologists, whereas it may comprise more than 50% of the work of most interventional radiologists. In view of pressing critical care engagements of cardiologists, the IAEA decided to introduce a simple programme of two days duration covering 12-14 hours of training. At the moment, such initiatives are not mandatory and it is desirable that the professional bodies should move further ahead in the best interest of the profession and professionals to implement the guidelines”.

## WEBSITE

IAEA has recently launched a public website specially dedicated to radiological protection of patients [1]. The website includes interventional cardiologists as one of target groups among health professionals. At the moment the website includes answers to the following questions:

**Patient protection**

- Are radiation induced skin injuries common among patients undergoing interventions?
- What problems are associated with diagnosis of such injuries?
- Can radiation injuries be prevented?
- How high is the exposure in cardiac interventions in comparison to chest radiograph?
- Which factors can affect patient dose in cardiac interventions?
- How can I manage patient exposure?
- What adverse effects could occur as a result of dose reduction actions?

**Staff protection** - Some of the measures to reduce patient dose will also result in a reduction of staff dose

- Is the exposure to the cardiologist much higher than to non-interventionalists?
- Is there a risk of developing cataracts after several years of work in a catheterization laboratory?
- Can I work my full professional life in a catheterization laboratory and have no radiation effects?

In future the website will have more information for patients. The information about training courses is available at the website [12].

## RESEARCH PROJECTS

Two coordinated research projects have just been completed, one entitled “Evaluate quantitatively and promote patient dose reduction approaches in interventional radiology” and another “A Pilot Study Exploring the Possibility of Establishing Guidance Levels in X ray Directed Interventional Procedures”. The results of both of these projects are under publication as TECDOCs of the IAEA and will provide useful material for cardiologists.

## ASSISTANCE TO MEMBER STATES THROUGH TECHNICAL COOPERATION (TC) PROJECTS.

The IAEA has regional projects in all the four regions of the world, namely Asia, Europe, Africa and Latin America. Information about these projects can be found at the website [13].

More than 30 countries are participating in Task 1, which involves radiation protection in interventional procedures. Results of these projects will be available on the above website in the near future

All these combined activities ensure that the IAEA plays a leading role in the world on radiation protection in cardiology.

## ACKNOWLEDGEMENT

The permission granted by the IAEA to publish this material is gratefully acknowledged.
